# A Comparison of Dynamic Strength Index between Team-Sport Athletes

**DOI:** 10.3390/sports5030071

**Published:** 2017-09-20

**Authors:** Christopher Thomas, Thomas Dos’Santos, Paul A. Jones

**Affiliations:** 1 Directorate of Sport, Exercise and Physiotherapy, University of Salford, Greater Manchester M6 6PU, UK; t.dossantos@edu.salford.ac.uk (T.D.); p.a.jones@salford.ac.uk (P.A.J.); 2 School of Health, Sport and Professional Practice, University of South Wales, Pontypridd CF37 1DL, Wales, UK

**Keywords:** peak force, countermovement jump, isometric mid-thigh pull, strength assessment

## Abstract

The purpose of this study was to examine the differences in countermovement jump peak force (CMJ-PF), isometric mid-thigh pull peak force (IMTP-PF), and resultant dynamic strength index (DSI) values between team-sport athletes. One hundred and fifteen male and female team-sport athletes performed the CMJ and IMTP to determine peak force (CMJ-PF and IMTP-PF, respectively). Statistically and practically significant differences (*p* ≤ 0.050; *d* = 0.49–1.32) in CMJ-PF were evident between teams. Specifically, the greatest CMJ-PFs were produced by the male cricket players and were followed in order by the male basketball, male soccer, female netball, female cricket, and female soccer players. Statistically and practically significant differences (*p* ≤ 0.045; *d* = 0.64–1.78) in IMTP-PF existed among sports teams, with the greatest IMTP-PFs were produced by the male soccer players and were followed in order by the male cricket, male basketball, female netball, female soccer, and female cricket players. Statistically and practically significant differences (*p* ≤ 0.050; *d* = 0.92–1.44) in DSI were found between teams. These findings demonstrate that CMJ-PF, IMTP-PF, and DSI differ between sports teams and provide normative data for ballistic and isometric PF measures. Strength and conditioning coaches should consider relative changes in CMJ-PF and IMTP-PF when assessing DSI ratios.

## 1. Introduction

Success in most sports is highly dependent on physical fitness characteristics, including strength and power. Furthermore, it is important to identify which mechanical variables dictate performance when designing, implementing, and monitoring training programs. Countermovement jump (CMJ) assessments are commonly used to monitor neuromuscular function [1] and can provide in-direct measures of lower-body power [2]. Likewise, the isometric mid-thigh pull (IMTP) is commonly used to safely measure one’s peak force (PF) and time-related force values [3]. Furthermore, both assessments are shown to strongly relate to sprinting [4], jumping [5], and change of direction performances [6,7]. Yet, the physical characteristics displayed by an individual may differ, depending on the sport, position, playing level, and training history of the athlete.

It has been previously observed that differences in physical characteristics are evident between starters and non-starters [8,9,10,11]. Likewise, previous research has established that physical characteristics differ between playing levels [12,13,14] and playing position [15,16,17]. However, with the exception of a few studies [18,19], very little attention has been paid to the differences in physical characteristics between sports teams. A recent study by Suchomel et al. [18] found differences in reactive strength index-modified (CMJ-RSImod) existed between six US collegiate sports teams during loaded and unloaded CMJ conditions. The findings revealed that collegiate sport teams exhibited different CMJ-RSImod characteristics during loaded and unloaded conditions. Specifically, men’s soccer and baseball produced the highest CMJ-RSImod values during unloaded conditions. Likewise, Sato et al. [19] found that significant differences in allometrically scaled IMTP-PF existed between US collegiate athletic teams. Again, the findings revealed men’s soccer and baseball to produce the greatest force output. However, none of these studies examined the differences in dynamic strength index (DSI) between sport teams. The DSI allows researchers and practitioners to determine the dynamic force capabilities of an athlete in relation to their maximum-force capability, and is expressed as a ratio of ballistic PF: isometric PF [20,21]. Thus, the DSI may be used as a means of assessing an athlete’s lower-body strength qualities and guide future training interventions [20].

The literature provides normative data for DSI ratios in various populations such as surfing [5,22,23,24], soccer and rugby league [25], collegiate [26], and mixed sample [20] athletes. Previous work by Sheppard et al. [20] recommended that a DSI ratio of <0.60 would be indicative of a need to increase ballistic strength training, while a DSI ratio of >0.80 would direct training toward the development of maximum strength. Specifically, mean DSI values range from 0.65 to 0.76 for surfers [5,22,23,24], while mean values for soccer and rugby league player are found to range from 0.82 to 0.84 [25]. Also, collegiate athletes mean DSI values are reported to be 0.78 [26], while Sheppard et al. [20] found mean DSI values of 0.70. This discrepancy could be attributed to inconsistencies in the methods of assessing ballistic PF, whereby some studies used the squat jump (SJ) [20,26], while others used the CMJ [5,22,23,24]. Indeed, a recent study by Comfort et al. [25] concluded that although no significant differences were found between DSI ratios calculated using SJ-PF or CMJ-PF, DSI calculated using CMJ-PF may provide a more reliable and less variable measurement, compared to SJ-PF.

Therefore, the aim of this study was to compare the CMJ-PF, IMTP-PF, and resultant DSI values between team-sport athletes. Based on the findings of previous research [18,19], it was hypothesized that statistically and practically significant differences in CMJ-PF, IMTP-PF, and DSI would exist among sports teams.

## 2. Materials and Methods

### 2.1. Subjects

This study included 115 male (*n* = 56) and female (*n* = 59) team-sport athletes. The male athletes participated in basketball (*n* = 17; height = 187.1 ± 9.4 cm, body mass 81.6 ± 10.5 kg), cricket (*n* = 23; height = 175.8 ± 6.1 cm, body mass = 76.9 ± 13.3 kg) and soccer (*n* = 16; height = 179.1 ± 5.2 cm, body mass 76.0 ± 8.6 kg), whereas the female athletes participated in netball (*n* = 21; height = 1.74 ± 6.1 cm, body mass = 66.7 ± 5.1 kg), cricket (*n* = 23; height = 165.2 ± 9.2 cm, body mass = 61.5 ± 11.1 kg) and soccer (*n* = 15; height = 168 ± 7.2 cm, body mass 56.2 ± 6.3 kg). Each athlete was in the preseason phase of training during his or her participation in this study. The investigation was approved by the institutional review board, and all provided appropriate consent to participate, with consent from the parent or guardian of all players under the age of 18. The study conformed to the principles of the World Medical Association’s Declaration of Helsinki.

### 2.2. Procedures

Each subject visited the Human Performance Laboratory for a single testing session as part of an ongoing athlete monitoring program. On arrival, all subjects had their height (Stadiometer: Seca, Birmingham, UK) and body mass assessed (Seca Digital Scales, Model 707: Seca, Birmingham, UK) while in bare feet, measured to the nearest 0.1 kg and 0.1 cm, respectively. Subjects were required to abstain from training for 48 h before testing and asked to maintain a consistent fluid and dietary intake on the day of testing as they would for normal skills training. Before the start of testing, athletes performed a standardized warm-up of activation and mobilization exercises, including various bodyweight lunges and squats, followed by some low level plyometric drills, replicating the participant’s standardized warm-ups before training. Furthermore, standardized progressive warm-ups were applied before all tests to control potential variables and improve the reliability of all tests.

### 2.3. Countermovement Jump Testing

Prior to maximal trials, each subject performed two warm-up CMJs, one at 50% and one at 75% of the subjects’ perceived maximum effort, separated by 1 min of rest. For all CMJs, subjects were instructed to jump “as high and as fast as possible”, with the arms akimbo. Depth of the eccentric phase was self-selected by the subjects to maximize CMJ height and ecological validity. Subjects performed three trials, with 1 min of rest between trials.

Countermovement jump data were collected using a portable force platform sampling at 1000 Hz (Kistler Instrument Corporation, Winterthur, Switzerland, Model 9286AA, SN 1209740). The force platform was interfaced with a laptop to allow for direct measurement of force-time characteristics, and then analyzed using Bioware software (Version 5.11; Kistler Instrument Corporation, Winterthur, Switzerland) and applied to a customized Microsoft Excel spreadsheet (version 2016, Microsoft Corp., Redmond, WA, USA). Prior to the onset of the countermovement, subjects remained stationary on the force platform for one second to enable an accurate measurement of body weight. Vertical ground reaction force data were then averaged across the first second, and the onset of the countermovement was determined when this value was reduced by 5 SDs [27]. Instantaneous velocity of the center of mass (COM) was calculated by dividing vertical ground reaction force (excluding body weight) by body mass and then integrating the product using the trapezoid rule. The concentric phase of the CMJ was then defined as occurring between the instant at which velocity of the COM exceeded 0.01 m·s^−1^ and take-off. [28] The instant of take-off was defined as the point when the vertical ground reaction force descended past 20 N. Concentric peak force (CMJ-PF) was determined from the unfiltered force-time history [29]. The mean performance of the three CMJs was used for further analysis.

### 2.4. Isometric Mid-Thigh Pull Testing

Following 5 min of rest, IMTPs were performed. Isometric peak force was assessed during isometric mid-thigh pull (IMTP) testing, using a portable force platform sampling at 600 Hz (400 Series Performance Force Plate; Fitness Technology) [7]. For the IMTP, subjects obtained self-selected knee and hip angles (knee = 130–150°; hip = 140–160°) based on the reports of previous research [30]. For this test, an immovable, collarless steel bar was positioned at approximately mid-thigh, just below the crease of the hip, using a portable IMTP rig (Fitness Technology, Adelaide, Australia). Each subject was provided two warm-up pulls, one at 50% and one at 75% of the subjects’ perceived maximum effort, separated by 1 min of rest. Subjects performed three maximal IMTPs, with the instruction to pull against the bar with maximal effort as quickly as possible, and push the feet down into the force plate; this instruction has been previously found to produce optimal testing results [31]. Each maximal isometric trial was performed for 5 s, and all subjects were given strong verbal encouragement during each trial. Two minutes of rest was given between the maximal effort pulls.

The maximum force recorded from the force-time curve during the 5-s IMTP trial was reported as the PF. The mean performance of the three was used for further analysis.

The DSI was calculated by dividing CMJ-PF by IMTP-PF.

### 2.5. Statistical Analysis

Data are presented as mean ± SD. Normality of data was assessed by Shapiro-Wilk statistic, and homogeneity of variance was verified with Levene’s test. A series of one-way analysis of variance were conducted to analyse differences in CMJ-PF, IMTP-PF, and DSI between sports. Where significant differences were found, Bonferroni post hoc analyses were completed to detect differences between sports. The magnitude of differences between sports was also expressed as standardized mean difference (Cohen’s *d*, effect sizes (ESs)) [32] and based on the scale by Hopkins [33]. All statistical analyses were completed using SPSS version 23 (IBM, New York, NY, USA), and statistical significance was set at *p* ≤ 0.05.

## 3. Results

No statistically significant differences in the homogeneity of variance existed among teams within the Levene’s test and, thus, equal variances were assumed. Descriptive CMJ-PF, IMTP-PF, and DSI data for each team are displayed in Table 1.

Statistically and practically significant differences in CMJ-PF were found between teams. Countermovement jump PF was greater in male basketball players compared with those in the female netball (*p* = 0.003; *d* = 0.55), female cricket (*p* < 0.001; *d* = 1.15), and female soccer (*p* < 0.001; *d* = 1.32) players. Likewise, male cricket players produced greater CMJ-PF compared to those produced by the female netball (*p* = 0.050; *d* = 0.63), female cricket (*p* < 0.001; *d* = 1.13), and female soccer (*p* < 0.001; *d* = 1.22) players. Male soccer players produced greater CMJ-PF compared to those produced by the female netball (*p* = 0.022; *d* = 0.49), female cricket (*p* < 0.001; *d* = 1.06), and female soccer (*p* < 0.001; *d* = 1.23) players. Finally, female netball players produced greater CMJ-PF compared to those produced by both female cricket (*p* = 0.005; *d* = 0.61) and female soccer (*p* = 0.022; *d* = 0.86) players.

Statistically and practically significant differences in IMTP-PF were found between teams. Isometric mid-thigh pull PF was greater in male basketball players compared with those in both female cricket (*p* < 0.001; *d* = 1.28), and female soccer (*p* < 0.001; *d* = 0.90) players. Likewise, male cricket players produced greater IMTP-PF compared to those produced by the female netball (*p* = 0.029; *d* = 0.74), female cricket (*p* < 0.001; *d* = 1.37), and female soccer (*p* < 0.001; *d* = 1.03) players. Male soccer players produced greater IMTP-PF compared to those produced by the male basketball (*p* = 0.045; *d* = 0.64), female netball (*p* < 0.001; *d* = 1.13), female cricket (*p* < 0.001; *d* = 1.78), and female soccer (*p* < 0.001; *d* = 1.39) players. Finally, female netball players produced greater CMJ-PF compared to those produced by the female cricket (*p* = 0.003; *d* = 0.74) players.

Statistically and practically significant differences in DSI were found between teams. Male soccer players produced lower DSI values compared to those produced by both female netball (*p* = 0.050; *d* = 0.92) and female cricket (*p* = 0.009; *d* = 1.44) players.

## 4. Discussion

The purpose of this study was to compare differences in CMJ-PF, IMTP-PF, and DSI between sports teams. The results of the current study indicate that statistically and practically significant differences in CMJ-PF, IMTP-PF, and DSI exist among sports teams.

The results of the current study indicate that the greatest CMJ-PFs were produced by the male cricket players and were followed in order by the male basketball, male soccer, female netball, female cricket, and female soccer players. Small to large effect sizes indicate that sizeable differences in CMJ-PF exist among sports teams in this study. These findings are in agreement with those by Suchomel et al. [18], who found that differences in jump height, time to take-off, and resultant CMJ-RSImod existed between collegiate sports teams. The data of the current study appears to be similar to CMJ-PF values obtained from previous studies on surfing (1134–1962 N) [5,22,23,24] as well as soccer and rugby (1854 N) [25] athletes. These results are likely related to players possessing high levels of strength and power for the many times they are required to perform explosive movements such as jumping and accelerating, all of which are important during offensive and defensive movements in the sports investigated within the current study. Specifically, these jumping actions require high levels of force and efficient usage of the stretch-shortening cycle (SSC) to compete for a shot at the net and intercept passes in basketball and netball. Female netball players are likely to produce greater levels of CMJ-PF than female cricket and soccer players because of the “jump-oriented” nature of the sport, requiring near-maximum levels of strength and power production. Likewise, cricket players require high levels of lower-limb power (for which high levels of force is important), which is key for wicketkeepers, pace bowlers, slip catchers, fielders, and batters, whereas the SSC is an important characteristic of many cricket movements. For example, vertical jump height is suggested to be an important contributor to bowling peak velocity in pace bowlers. Furthermore, the optimal “ready” position for in-fielders closely resembles the position and postures seen in a CMJ, which may influence their performance in a testing setting. However, these differences may be partly explained by task specificity, whereby netball players may develop muscle strength qualities which reflect the characteristics of the sport. Furthermore, it could be argued that differences in CMJ-PF were due to sex differences, with research showing that differences in physical characteristics exist between females and males [34]. It may be the case therefore that sex plays a major role in differences in force production capabilities. This notion may be supported by the differences in IMTP-PF of the current study, whereby male players produced statistically and practically greater IMTP-PFs compared to female players.

The current study found that statistically and practically significant differences in IMTP-PF existed among sports teams. The greatest IMTP-PFs were produced by the male soccer players and were followed in order by the male cricket, male basketball, female netball, female soccer, and female cricket players. Moderate to large effect sizes indicate that sizeable differences in IMTP-PF exist among sports teams in this study. The IMTP-PF values of the current study appear to be in line with those obtained from previous research in surfing (1924–3038N) [5,22,23,24] and collegiate [26] athletes, but are lower compared to mixed sample (2879 N) [20] athletes. As previously mentioned, a possible explanation for this result might be due to sex differences, with research showing that differences in physical characteristics exist between females and males [18,34]. Another possible reason might be due to the different characteristics of the sport or playing positions. For example, basketball players perform approximately 1000 acyclical changes of activity, whereas soccer players perform 727 ± 203 change of direction maneuvers per game, requiring high levels of concentric, isometric, and eccentric strength to decelerate and re-accelerate in multiple planes of movement [35]. Therefore, the ability to apply and resist force over various types of loading to achieve high movement velocities is critical for performance. Also, high levels of strength are important for reducing the risk of injury. Peak vertical ground reaction forces for basketball athletes during landing tests has been shown to range from 3.53 to 5.74 × body weight (BW) [36], while lay-up landing forces in knee flexion have been shown to be double those during other common movements (running, shuffling, cutting) [37]. Similarly, ground reaction forces were reported to be 9 × BW [38] during the front-foot strike of the delivery stride in pace bowlers. Also, previous research has found that differences in physical characteristics exist between positions [15,17]; therefore, this may have influenced the findings of the current study. Sato et al. [19] found that differences in IMTP-PF existed among collegiate sports teams. Specifically, male soccer players produced the greatest IMTP-PF values, and were followed in order by male baseball, male tennis, female volleyball, female soccer, and female tennis players. The findings of the current study are similar in that male soccer players produced the greatest IMTP-PF values, followed by male cricket players. These findings may indicate that although soccer and cricket require sport-specific skills to be successful, high levels of isometric strength may be vital to their overall playing ability. Indeed, research has shown IMTP-PF to strongly relate to sprint [7], jump [39], and change of direction abilities [7] in these populations.

Another important finding was that statistically and practically significant differences in DSI existed among sports teams. Specifically, the lowest DSI values were produced by the male soccer players, and were followed in order by the female soccer, male basketball, male cricket, female netball, and female cricket players. Male soccer DSI values appear to be lower than previously reported in a sample consisting of soccer players (0.84) [25]. However, the sample of Comfort et al. [25] consisted of soccer and rugby league athletes; therefore, the specific DSI ratio of soccer players only is unknown. It is interesting to note that no statistically or practically significant differences in DSI existed between sports teams, except between the male soccer players and both the female netball and female cricket players. These findings suggest that differences may be due to sex differences and not sport differences, especially since no statistically or practically significant differences were observed between male soccer and basketball players. These results further support the idea that athletes’ strength levels cannot be distinguished solely on their DSI ratio. For example, female soccer players were ranked sixth and fifth for CMJ-PF and IMTP-PF, respectively, while according to DSI value, female soccer players were ranked second. It can therefore be assumed that although the DSI can be used to assess an athlete’s dynamic force capabilities in relation to their maximum-force capability, it is important to focus on the relative effect of changes in CMJ-PF and IMTP-PF, as the ratio can change in response to different phases of resistance training [20].

The major limitation of this study is the absence of comparisons between positions within each sport. Previous research indicates that physical characteristics differ among playing position; therefore, it would be interesting to assess whether differences in CMJ-PF, IMTP-PF, and the resultant DSI values are evident among cohorts similar to those assessed in the current study. Second, this study did not examine the influence of physical maturation on CMJ-PF and IMTP-PF. Research has shown that physical capabilities develop in a nonlinear fashion because of growth and maturation, which may have affected the findings in the current study.

## 5. Conclusions

This study set out to compare CMJ-PF, IMTP-PF, and the resultant DSI values between sports teams. This study has shown that statistically and practically significant differences in CMJ-PF, IMTP-PF, and DSI exist between sports teams. Based on the results of the current study, it is suggested that athletes’ strength levels cannot be distinguished solely on their DSI ratio, and researchers and practitioners should pay close attention to relative changes in CMJ-PF and IMTP-PF.

## Figures and Tables

**Table 1:** Descriptive team data for ballistic and isometric variables.

Team	CMJ-PF (N) ^‡^	IMTP-PF (N) ^‡^	DSI ^†^
M Basketball	1952 ± 207	2297 ± 360	0.86 ± 0.13
M Cricket	2056 ± 559	2469 ± 674	0.86 ± 0.21
M Soccer	1943 ± 262	2853 ± 581	0.70 ± 0.16
F Netball	1651 ± 239	1925 ± 374	0.89 ± 0.22
F Cricket	1351 ± 269	1495 ± 305	0.91 ± 0.13
F Soccer	1328 ± 290	1697 ± 268	0.80 ± 0.23

Note: CMJ = countermovement jump; IMTP = isometric mid-thigh pull; PF = peak force; DSI = dynamic strength index; M = male; F = female. ^†^ Statistically significant difference between teams (*p* ≤ 0.05); ^‡^ Statistically significant difference between teams (*p* ≤ 0.001).
